# Assessment of accuracy, fix success rate, and use of estimated horizontal position error (EHPE) to filter inaccurate data collected by a common commercially available GPS logger

**DOI:** 10.1371/journal.pone.0189020

**Published:** 2017-11-30

**Authors:** Gail Morris, L. Mike Conner

**Affiliations:** Joseph W. Jones Ecological Research Center, Jones Center Dr., Newton, Georgia, United States of America; Public Library of Science, FRANCE

## Abstract

Global positioning system (GPS) technologies have improved the ability of researchers to monitor wildlife; however, use of these technologies is often limited by monetary costs. Some researchers have begun to use commercially available GPS loggers as a less expensive means of tracking wildlife, but data regarding performance of these devices are limited. We tested a commercially available GPS logger (i–gotU GT–120) by placing loggers at ground control points with locations known to < 30 cm. In a preliminary investigation, we collected locations every 15 minutes for several days to estimate location error (LE) and circular error probable (CEP). Using similar methods, we then investigated the influence of cover on LE, CEP, and fix success rate (FSR) by constructing cover over ground control points. We found mean LE was < 10 m and mean 50% CEP was < 7 m. FSR was not significantly influenced by cover and in all treatments remained near 100%. Cover had a minor but significant effect on LE. Denser cover was associated with higher mean LE, but the difference in LE between the no cover and highest cover treatments was only 2.2 m. Finally, the most commonly used commercially available devices provide a measure of estimated horizontal position error (EHPE) which potentially may be used to filter inaccurate locations. Using data combined from the preliminary and cover investigations, we modeled LE as a function of EHPE and number of satellites. We found support for use of both EHPE and number of satellites in predicting LE; however, use of EHPE to filter inaccurate locations resulted in the loss of many locations with low error in return for only modest improvements in LE. Even without filtering, the accuracy of the logger was likely sufficient for studies which can accept average location errors of approximately 10 m.

## Introduction

Global positioning system (GPS) devices can substantially increase the amount of data that can be collected when used to monitor wildlife and are replacing VHF (very high frequency) radio telemetry as the preferred means of tracking medium and large–sized wildlife species. However, one major drawback to using GPS devices is the monetary expense, which is substantially greater per device than VHF radio transmitters. Recently, wildlife researchers have begun to try to strike a balance between the benefits of increased data collection and the expense of GPS devices produced by wildlife telemetry companies by using commercially available GPS loggers which are designed for tracking pets and recording travel and exercise routes. Two of these devices, the i–gotU GT–120 (Mobile Action Technology, Inc., Taiwan) and the nearly identical CatTraQ (Perthold Engineering, LLC, USA) have particularly found use in seabird research (black-legged kittiwake, *Rissa tridactyla* [1]; Atlantic puffins, *Fratercula arctica* [2]; Northern gannets, *Morus bassanus* [3]; shearwaters [4]; wandering albatross, *Diomedea exulans* [5]; yellow-legged gulls, *Larus michahellis* [6]; and little penguins, *Eudyptula minor* [7]). They have also been used to monitor a variety of other species including feral cats (*Felis catus* [8, 9]), Eastern gray squirrels (*Sciurus carolinensis* [10]), pigeons (*Columba livia* [11]), European badgers (*Meles meles* [12]), and Irish hares (*Lepus timidus hibernicus* [13]), and have found use in veterinary and human health research [14, 15, 16]. Drawbacks of these devices include the lack of a drop–off mechanism and short battery life, but pairing the loggers with radio transmitters and/or replacing the loggers’ batteries with larger batteries can mitigate these problems [10, 13, 17].

Despite the increasing number and diversity of studies using commercially available GPS loggers, there have been few rigorous evaluations of their performance. Studies which have done so have used stationary [18, 19, 20] and motion controlled tests [19, 20] in varying habitats [20] and sky-view conditions [18, 20] to quantify location error (LE, distance between the true and estimated location) and fix success rate (FSR, proportion of times a logger attempts a fix and is successful). These studies have found LE and FSR comparable to GPS loggers produced by wildlife telemetry companies. Despite these promising findings, one persistent drawback to use of these loggers is the lack of an estimate of potential error associated with fixes.

GPS devices designed by wildlife telemetry companies typically record the number of satellites used to fix each location and an estimate of satellite number and position relative to the GPS device (dilution of precision, DOP). Dilution of precision is negatively correlated with LE and may be used to identify locations which have the potential for inaccuracy [21, 22, 23]. However, using DOP to filter locations to improve LE typically also results in the loss of many locations with acceptable error [21, 23, 24]. Location error is also inversely related to number of satellites used to record a location [23], particularly when comparing 2 dimensional (2-D, uses 3 satellites to fix a location) and 3 dimensional locations (3-D, uses 4 or more satellites to record a location [25, 26]). On average, 3-D locations are more accurate than 2-D locations and some researchers have suggested taking this into account when screening locations by recommending 2-D and 3-D locations be screened separately and with greater rigor applied to 2-D locations [21].

Estimated horizontal position error (EHPE, the radius of a circle around the actual location which is estimated to contain the GPS-estimated location with 1 sigma (approximately 68%) of uncertainty), an output provided by devices using SiRF star III chipsets (e.g., i–gotU, CatTraQ, and CatLog GPS loggers), may be used to filter locations with potentially high LE [17]. Such a measure of error would have clear benefits for data analyses. Therefore, the objectives of our study were threefold. First, we sought to add to the body of literature providing quantitative estimates of the accuracy and FSR of i-gotU GT-120 GPS loggers in optimum conditions and varying degrees of cover so that researchers may be able to make informed decisions regarding whether these loggers are suitable for their research needs. Second, we examined the relationship between EHPE and number of satellites and LE as this has, to our knowledge, not previously been reported but is necessary to determine whether EHPE has utility in the identification of potentially inaccurate locations. Finally, we used EHPE as a filter to remove potentially inaccurate locations and quantified how doing so, at varying levels of EHPE, influenced the mean LE of remaining locations and the proportion of data lost.

## Methods

All trials were carried out at the Joseph W. Jones Ecological Research Center at Ichauway, in Baker County, Georgia, USA (hereafter, Ichauway). Several ground control points (GCP) are established on Ichauway. GCPs are permanent locations (here, survey markers cemented into the ground) whose locations are known with great precision and which are used to geo–reference aerial photo data [27]. GCP locations on Ichauway are known to within 30 cm and, as GCPs are meant to be easily seen in aerial photos, are in areas with little to no canopy cover. The topography of the region is very flat.

### GPS loggers and supplemental data acquisition

We used 4 i–gotU GT–120 GPS loggers for our trials. The i–gotU GT–120 GPS loggers use a SiRF III chipset, a built-in patch antenna, 20 channels, and a sensitivity of -159 dBm. The current cost is US$45–70, and the device weighs 22 g. For purposes of comparison, the CatTraQ (variously marketed as CatTrack1 and CatLog) has the same specifications, approximate cost, and weight. The i–gotU GT-600 (Mobile Action Technology, Inc., Taiwan) also has similar specifications, with the exception of a larger battery and therefore costs US$65–85 and weighs 37 g.

The output containing EPHE values for each location is not included with the primary data download, nor is it documented in the device user manual. This data can be accessed by creating a folder titled GT_DATA_LOG on the local drive of the computer used to download the GPS data (Mobile Action Technologies representative Bryan Huang, personal communication). The file downloaded into this folder contains additional data associated with each location including EHPE (in cm) and number of satellites used to fix the location. Users must re-name or move the file if they wish to avoid overwriting previous data downloads.

### Preliminary trials

Preliminary data collection took place in March and December of 2014 with the objective of quantifying the accuracy of the loggers in optimum conditions (i.e., flat topography and minimal overhead cover). In March, we set 1 i–gotU GT–120 logger at each of 2 GCPs. Previous research has shown that antenna angle and fix interval may influence accuracy of GPS loggers [20]. It was not our intent to test either factor, but we ensured that variations in antenna angle and fix interval would not influence our results by keeping these factors consistent each time a logger was set. The loggers were placed in plastic bags for waterproofing and were set on the GCP (horizontally, at ground level). Loggers were programmed to collect a location every 15 minutes over 2 to 3 days. Studies which have used the i-gotU GT-120 and similar loggers collected data at intervals ranging from 5 s to 30 min [1–16], making 15 minutes a reasonable interval for assessment of this logger. We used a minimum of 2 days for data collection because we felt this would provide an adequate number of locations for our analyses (the minimum number of locations collected per trial was 174). This duration of data collection also ensured that each session included multiple satellite configuration cycles. Variations in duration of data collection were merely due to convenience.

In December, we expanded our sample size by collecting data from an additional 6 deployments of i–gotU GT–120 loggers following the same procedures. During the December session, we collected data at the 2 GCPs at which data were collected in March as well as at 4 new GCPs (i.e., over 8 total deployments, we collected data at 6 GCPs, 2 of which were used twice). Additionally, during the December session, we used the 2 loggers which were tested in March as well as 2 additional loggers of the same type (i.e., between the March and December sessions, we tested 4 total loggers, and each was deployed twice).

### Cover trials

**Fig 1:**
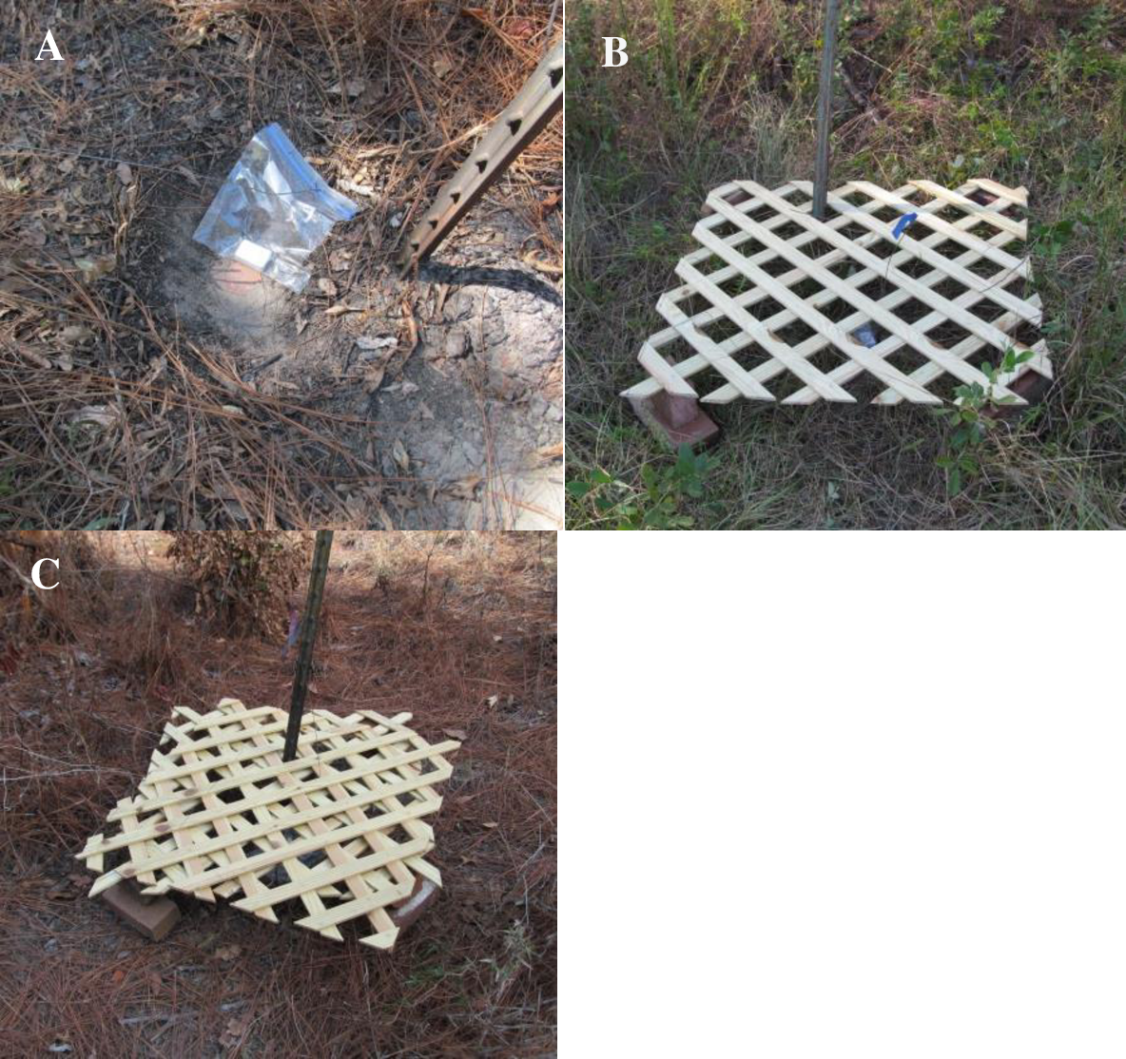
Set-up of trials to determine effect of cover on accuracy of i-gotU GT-120 GPS loggers. Cover treatments included A) no cover, B) logger centered under a single layer of wooden lattice, and C) logger centered under two layers of lattice, offset. The GPS loggers were set at ground control points (GCPs) with accuracy known to ≤ 30 cm in Baker County, Georgia, USA in October 2015.

In October of 2015, we conducted a second trial with the intent of quantifying accuracy of the loggers at multiple levels of cover. Because the GCPs were by definition in open areas (because, when established, they were intended to be easily seen in aerial photos), we constructed artificial cover at the GCPs. This also enabled us to standardize the cover treatment levels across GCPs. We used the same 4 loggers as in the preliminary trials and they were set following the same protocols, except that the loggers collected data over 3 to 4 days for each level of cover. Each logger was set at one GCP for 3 cover treatments: 1) no cover, 2) centered under a single layer of 80 cm x 80 cm wooden lattice, and 3) centered under 2 layers of wooden lattice with the upper layer slightly offset (Fig 1). The lattice was supported at the corners by two bricks so that the lattice was approximately 15 cm above the ground. We used overhead photographs of the lattice treatments and the ImageJ image processing program [28] to calculate the percent area of the lattice in each treatment to estimate percent overhead cover. There was approximately 60% overhead cover in the single lattice treatment and approximately 80% cover in the double lattice treatment. When switching from one cover treatment to another, loggers were collected and the data were downloaded before resetting the cover treatment and the logger (i.e., the logger had to reacquire satellites for each cover treatment level). For the cover tests, we recorded the time loggers were turned on and picked up which enabled us to quantify FSR. To prevent confounding of cover treatment levels with date, cover treatments were staggered so that sites received different treatments on different days.

### Statistical methods

For both the preliminary and cover trial datasets, we used ArcGIS 9.3.1 [29] to find the distance from each recorded location to the GCP (i.e., the LE for each fix). For both trials, we summarized accuracy of the loggers by calculating mean LE, median LE, and 50 and 95% circular errors probable (CEP, the radius of a circle which includes a specified percentage of locations) for each deployment. For the cover trials, we also calculated FSR for each trial (these data were not available for the preliminary trials).

We conducted several analyses of the location data. First, to determine whether the cover treatment influenced performance of the loggers we used linear mixed effects models implemented in the MIXED procedure in program SAS version 9.3 (SAS Institute, Inc., Cary, NC, USA) to separately model 1) LE and 2) FSR as a function of cover treatment. For the model evaluating effect of cover on LE, LE was log-transformed to meet normality assumptions. For both models, site/device (the two were confounded because, for the cover trials, each logger was used at a single site) was specified as a random effect while the cover treatment was the only fixed effect. If the treatment effect was found significant, multiple comparisons between treatment levels were made using Scheffé’s method to determine which treatment levels differed significantly from each other. For these and all analyses, results were considered significant at α ≤ 0.05. Normality assumptions were evaluated by visual inspection of normal probability plots and plots of residuals vs. predictions.

Second, to determine whether LE can be predicted by EHPE or number of satellites, we combined all location data (from preliminary and cover trials) and used linear mixed-effects models implemented in the MIXED procedure in program SAS to model LE as a function of 1) EHPE and 2) number of satellites. We used set (each set of data collected by a logger over a single data collection session) as a random effect and log-transformed LE to meet normality assumptions for both models. EHPE and number of satellites were each specified as the only fixed effect in their respective models. Because our models included a random effect, we were not able to evaluate the portion of variation in LE explained by EHPE or number of satellites using the standard R^2^ estimate. Instead, we calculated marginal and conditional *R*^2^ values (*R*^2^_GLMM(*m*)_, refers to variance explained by fixed effects alone, and *R*^2^_GLMM(*c*)_, refers to variance explained by both fixed and random effects, respectively; [30]) for each model using the MuMin package in program R version 3.1.1 [31, 32].

Finally, again using the combined data set, we assessed EHPE as a filter to improve LE by setting 8 EHPE thresholds. The first 5 thresholds were set at intervals of 500 cm. Because the EHPE data were heavily skewed, the next 3 thresholds were set at wider intervals (EHPE ranged from 240 to 32512 cm with a mean of 2329 cm). Use of these thresholds allowed presentation of what we considered a reasonable range data retention. For each, we removed all locations with EHPE values above the threshold, calculated the percentage of locations removed, and summarized LE and CEP of remaining locations.

## Results

### Preliminary trials

We collected 1,874 locations from the 8 preliminary deployments. The mean LE was 11.6 m (± SE 0.5 m) with a median LE of 4.9 m and a range from 0.1 to 529.7 m. Of all locations, 88.4% were within 25 m of the GCP, 95.3% were within 50 m, and 99.5% were within 100 m. The mean 50% CEP was 8.4 m (± 3.6 m) and the mean 95% CEP was 28.4 m (± 8.8 m).

### Cover trials

We collected a total of 3827 locations during the cover trials. From the no cover treatment, we collected 1166 locations with a mean LE of 6.68 m (± 0.26 m), a median LE of 4.56 m, and a range of 0.29 to 107.72 m (Table 1). From the single lattice treatment, we collected 1266 locations with a mean LE of 8.35 m (± 0.51 m), a median LE of 4.79 m, and a range of 0.25 to 372.02 m. From the double lattice treatment, we collected 1395 locations with a mean LE of 8.92 m (± 0.47 m), a median LE of 4.94 m, and a range of 0.25 to 381.30 m.

**Table 1:** Mean and median location error (LE), fix success rate (FSR), percents of locations at three location error thresholds, and circular errors probable (CEP) of locations collected by i–gotU GT–120 GPS loggers in 3 cover treatments. Cover treatments included either 1 or 2 layers of wooden lattice (see Fig 1), and a control with no cover. Data were collected in Baker County, Georgia, USA in October 2015.

Treatment	Mean LE ± SE (m)	Median LE (m)	FSR ± SE (%)	% LE < 25m	% LE < 50m	% LE < 100m	50% CEP ± SE (m)	95% CEP ± SE (m)
No cover	6.68 ± 0.26	4.56	101.3 ± 0.78	97.3	99.2	99.8	5.43 ± 0.44	19.57 ± 3.72
1x lattice	8.35 ± 0.51	4.79	101.2 ± 0.61	95.2	98.6	99.6	5.13 ± 0.47	24.19 ± 2.63
2x lattice	8.92 ± 0.47	4.94	99.6 ± 1.36	93.9	98.1	99.4	5.79 ± 0.45	27.41 ± 4.01

LE was significantly influenced by the cover treatment (*P* < 0.001, *F*_2,3821_ = 9.64). Multiple comparisons between treatment levels indicated that the no cover and single lattice treatments had similar magnitudes of LE (*P* = 0.95), while the double lattice treatment had significantly greater LE than both the no cover and single lattice treatments (*P* = 0.002 for both comparisons). Greater degrees of cover were associated with lower FSR, but in all cover treatments FSR was ≥ 100% (Table 1; although the loggers were programmed to attempt a fix every 15 minutes, at times the actual interval between fixes was slightly less than 15 minutes), and differences between treatments were not significant (*P* = 0.46, *F*_2,6_ = 0.93).

### Relationships between EHPE, number of satellites, and location error

**Fig 2:**
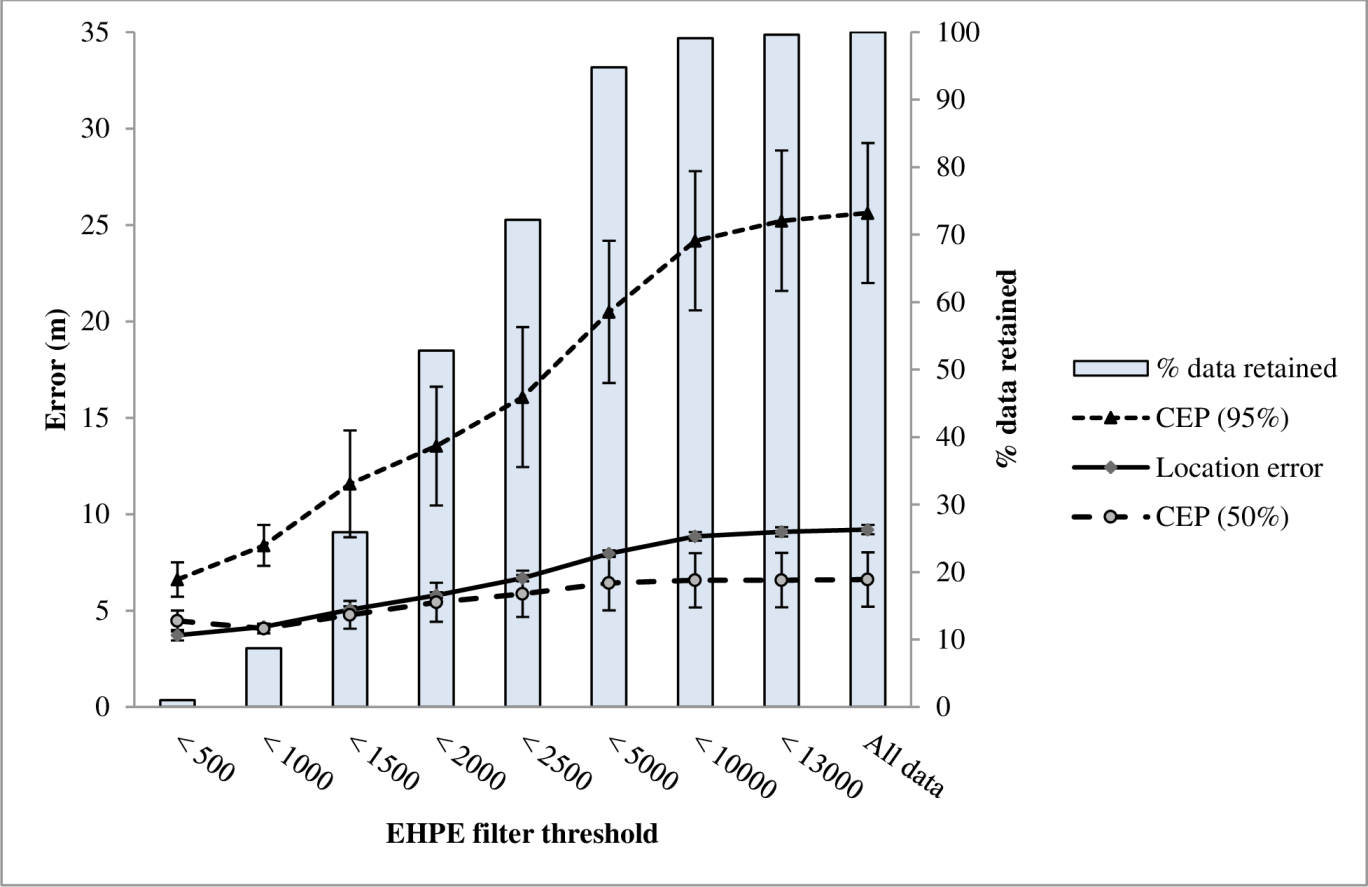
Mean location error (LE, ± SE) and 50 and 95% circular errors probable (CEP, ± SE) at 8 thresholds of estimated horizontal position error (EHPE) used to filter data, with percent data retained at each filter threshold. Data were collected by i–gotU GT–120 GPS loggers in Baker County, Georgia, USA in March and December, 2014 and October 2015.

**Fig 3:**
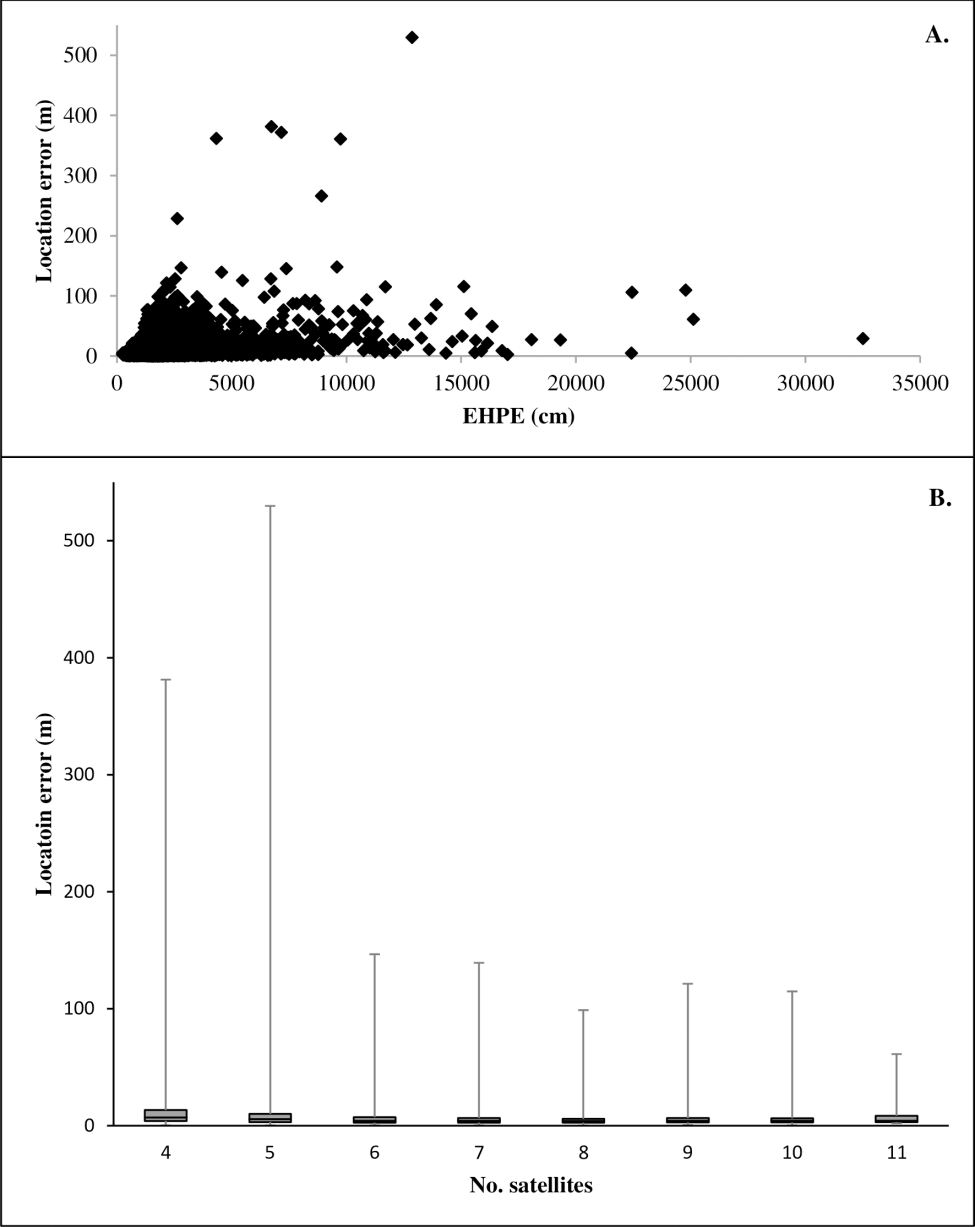
**A) Scatterplot showing the relationship between location error and estimated horizontal position error (EHPE) and B) box and whiskers plot showing relationship between number of satellites and EHPE.** Data were collected in Baker County, Georgia, USA in March and December 2014 and October 2015.

The combined preliminary and cover trials data set included 5701 locations. In this data set, mean LE was 9.2 m (± SE 0.2 m, with a median of 4.84 m and a range from 0.1 to 529.7 m; Fig 2). Visual inspection of the data showed a weak relationship between LE and both EHPE and number of satellites (Fig 3). The linear mixed-effects models showed that both EHPE and number of satellites were significant predictors of LE (EHPE, *P* < 0.001, *F*_*1*,*5680*_ = 1056.7; number of satellites, *P* < 0.001, *F*_*1*,*5680*_ = 338.5). For the model examining the influence of EHPE on LE, the *R*^2^_GLMM(*m*)_ and *R*^2^_GLMM(*c*)_ values were 0.13 and 0.31, respectively. For the model examining influence of number of satellites on LE, the *R*^2^_GLMM(*m*)_ and *R*^2^_GLMM(*c*)_ values were 0.05 and 0.26, respectively.

### EHPE as a filter to improve location error

When examining use of EHPE to filter locations, we found a positive relationship between threshold values of EHPE and mean LE and CEP (Fig 2). However, use of EHPE to filter data resulted in the loss of a large number of locations for modest improvements in error (Fig 2, Table 2). Sample sizes for Fig 2 and percentages of locations with LE < 25, 50, and 100 m are shown in Table 2.

**Table 2:** Percent of locations with location error < 25, 50, and 100 m at 8 thresholds of estimated horizontal position error (EHPE) used to filter data collected by i–gotU GT–120 GPS loggers. Data were collected in Baker County, Georgia, USA in March and December, 2014 and October 2015.

EHPE (cm)	*n*	% locations with error <			% data retained
		25 m	50 m	100 m	
All	5701	93.1	97.5	99.6	100
< 13,000	5678	93.3	97.6	99.6	99.6
< 10,000	5648	93.6	97.8	99.7	99.1
< 5,000	5407	94.7	98.3	99.8	94.8
< 2,500	4118	96.2	98.7	99.9	72.2
< 2,000	3009	97.1	99.1	100	52.8
< 1,500	1476	97.8	99.5	100	25.9
< 1,000	495	99.6	100	100	8.7
< 500	55	100	100	100	1.0

## Discussion

We quantified LE and FSR of a common commercially available GPS logger, the i-gotU GT–120, in optimum conditions and in varying levels of cover and assessed whether error could be improved by filtering locations using EHPE. Using our full dataset (including locations from all trials, with and without added cover), LE averaged 9.2 m (± 0.2 m) and the mean 50% CEP was 6.6 m (± 1.4 m) (Fig 2). This is similar to, or better than, estimates reported by other researchers with the same or similar devices. Duncan et al. [18] reported a mean LE of 19.6 m and a 50% CEP of 10.8 m (estimated from stationary loggers set at sites varying in sky availability) for the i–gotU GT–600. Forin-Wiart et al. reported a mean LE of 15.4 m using stationary CatLog GPS loggers set in a variety of habitat conditions [20]. Vazquez-Prokopec et al. measured a root mean squared error (RMSE) of 4.4 m in stationary tests and 10.3 m in linear path tests using i-gotU GT-100 loggers [19]. In less rigorous tests, Reid and Harrison [13] reported LE of the i–gotU GT–120 at 38.8 m and Dürr and Ward [14] reported locations collected by stationary CatTraQ loggers averaged 18.3 m from their centroid. In comparison, the mean range of measurement error reported by users of GPS devices produced by 4 wildlife telemetry companies ranged from 10 to 60 m [22].

Addition of cover caused accuracy to decline, although the mean LE was still < 10 m even in our highest cover treatment. Although our analysis found that locations from the densest cover treatment had higher LE than locations with no or lower levels of cover, the magnitude of the differences between treatments was small; the difference in mean LE between the no cover treatment and the double lattice treatment was 2.2 m, which is unlikely to have much importance in the context of data collected from wildlife. Similar, but more dramatic, results have been reported with other commercially available GPS loggers ([18, 20], i–gotU GT–600 and CatLog loggers, respectively). Both Duncan et al. [18] and Forin-Wiart et al. [20] found significant differences in LE with LE increasing with increasing sky obstruction. We were not able to make comparisons of degree of cover between this and other studies; however, it is possible that even our highest level of cover may have been less severe than the cover in other studies. This may explain the small increase in LE we observed in response to increasing cover, compared to larger magnitudes of LE in other studies [18, 20].

This study found FSR was near 100%, even in our densest cover treatment and was often > 100%. This occurred because, although we specified a data collection interval of 15 minutes, locations were often collected at intervals just under 15 minutes. Other researchers have observed similar results (FSR > 100%) using the CatLog GPS logger [20] and the i-gotU GT-120 [17]. Our highest cover treatment had little effect on FSR. Forin-Wiart et al. [20] similarly found no effect of canopy cover on FSR, but observed that habitats that completely obscured a portion (logger placed next to a wall) or all (logger placed inside a house) of the sky experienced reduced FSR (75% and 20% FSR, respectively), with FSR declining with declining sky-view, which has clear implications for use of these loggers to track species which use especially dense habitats, spend time underground, or live in areas with steep topography. However, this is a concern even among GPS loggers produced by wildlife telemetry companies and in both our and the Forin-Wiart et al. study [20], FSR values were comparable to those observed with GPS loggers produced by wildlife telemetry companies.

We found a significant positive relationship between EHPE and LE and a significant negative relationship between number of satellites and LE. However, our investigation into whether EHPE may be useful in filtering positions with high LE suggests that doing so has limited utility. Use of low to moderate EHPE thresholds caused significant loss of data in return for only modest improvements in mean LE. Filtering by EHPE appears most useful to remove locations with very high LE, although doing so caused loss of locations with low error and did not remove all locations with high errors. On our study site, setting an EHPE filter threshold of 5000 cm would allow retention of 95% of locations while eliminating most locations with large errors (Figs 2 and 3). We suggest that researchers could benefit from conducting tests to identify optimum EHPE threshold values specific to their study sites and habitats likely to be frequented by their target species. The relationship between LE and EHPE may vary depending on factors such as degree of cover or topography. Additionally, since fix interval influences LE [20], researchers should conduct such tests using the fix interval anticipated during deployment on research animals. Although it was beyond the scope of our investigations, future research may benefit from examining the potential influence of fix interval on the utility of EHPE as a filter for inaccurate locations.

EHPE is not unlike DOP measures often used to filter GPS data. Horizontal dilution of precision (HDOP) and positional dilution of precision (PDOP) explain only 21 to 26% of the variation in LE [21, 23, 24]. Researchers examining error in relation to DOP similarly have found that filtering locations based on DOP estimates removes some locations with high error but at the cost of many locations with acceptable error. Therefore, we suggest that recommendations for handling data with respect to DOP are likely to apply to EHPE as well (for example, see [21, 23, 24]). Recently, Laver et al. [33] suggested evaluation of multiple screening metrics (i.e., DOP, number of satellites, fix dimension (2D or 3D), estimated elevation error) in an information-theoretic framework to determine which screening metrics are best-suited for screening data from individual study sites. For data collected from GPS loggers using SiRF iii chipsets, EHPE is likely to provide an additional metric for such evaluation.

Screening methods based on animal behavior provide an alternative for identifying erroneous locations. For example, with knowledge of the speed at which a study animal is able to travel, it is possible to identify and remove locations which suggest improbable or impossible travel distances given time between locations [1, 13, 14]. The nonmovement method developed by Bjørneraas et al. [34] screens unrealistic locations by identifying those which would have required an animal to exceed specified thresholds for distance, speed, and turning angles between locations. When applied to moose (*Alces alces*) GPS data, the nonmovement method performed better than DOP-based screening methods to remove obviously erroneous locations while minimizing data loss [34]. However, this method performs best with relatively short data collection intervals [34].

We conclude that EHPE and number of satellites have a statistically significant relationship with LE but that EHPE provides limited benefit in filtering imprecise locations collected by the i–gotU GT–120 GPS logger. This is likely also true of the nearly identical CatTraQ and CatLog GPS loggers which record the same information as the i–gotU GT–120 and which is accessible in the same fashion; however, we did not include either of these devices in our tests. Researchers who wish to use EHPE as a filter for potentially inaccurate locations should conduct preliminary investigations to identify site-specific EHPE thresholds for acceptable data loss relative to improvements in mean LE. Such preliminary investigations also aid in understanding how habitats and topography in a study area may influence LE and FSR and how these variables may introduce bias in data collection [22, 23]. Given the cost of commercially available GPS loggers is substantially less than GPS devices produced by wildlife telemetry companies while LE and FSR values are comparable, they represent a promising tool for wildlife researchers.

## Supporting information

S1 Appendix Location error, number of satellites, and estimated horizontal position error (EHPE) values for locations collected by i-gotU GT-120 GPS loggers in Baker County, Georgia, in 2014 and 2015. (XLSX)

S2 Appendix Circular errors probable (CEP, 50 and 95%) calculated for each of 20 GPS data collection sessions at 8 thresholds of estimated horizontal position error (EHPE). Data were collected using i-gotU GT-120 GPS loggers in Baker County, Georgia in 2014 and 2015.
(XLSX)

S3 Appendix Fix success rates for each i-gotU GT-120 GPS logger deployment during trials assessing effects of cover on logger performance. Data were collected in Baker County, Georgia in 2015.
(XLSX)
